# Neuroanatomy goes viral!

**DOI:** 10.3389/fnana.2015.00080

**Published:** 2015-07-01

**Authors:** Jonathan J. Nassi, Constance L. Cepko, Richard T. Born, Kevin T. Beier

**Affiliations:** ^1^Systems Neurobiology Laboratories, Salk Institute for Biological StudiesLa Jolla, CA, USA; ^2^Department of Genetics, Harvard Medical SchoolBoston, MA, USA; ^3^Department of Ophthalmology, Howard Hughes Medical Institute, Harvard Medical SchoolBoston, MA, USA; ^4^Department of Neurobiology, Harvard Medical SchoolBoston, MA, USA; ^5^Center for Brain Science, Harvard UniversityCambridge, MA, USA; ^6^Department of Psychiatry and Behavioral Sciences and Department of Biology, Stanford UniversityStanford, CA, USA

**Keywords:** neurotropic virus, neuroanatomy, review, transsynaptic tracing, expression vectors

## Abstract

The nervous system is complex not simply because of the enormous number of neurons it contains but by virtue of the specificity with which they are connected. Unraveling this specificity is the task of neuroanatomy. In this endeavor, neuroanatomists have traditionally exploited an impressive array of tools ranging from the Golgi method to electron microscopy. An ideal method for studying anatomy would label neurons that are interconnected, and, in addition, allow expression of foreign genes in these neurons. Fortuitously, nature has already partially developed such a method in the form of neurotropic viruses, which have evolved to deliver their genetic material between synaptically connected neurons while largely eluding glia and the immune system. While these characteristics make some of these viruses a threat to human health, simple modifications allow them to be used in controlled experimental settings, thus enabling neuroanatomists to trace multi-synaptic connections within and across brain regions. Wild-type neurotropic viruses, such as rabies and alpha-herpes virus, have already contributed greatly to our understanding of brain connectivity, and modern molecular techniques have enabled the construction of recombinant forms of these and other viruses. These newly engineered reagents are particularly useful, as they can target genetically defined populations of neurons, spread only one synapse to either inputs or outputs, and carry instructions by which the targeted neurons can be made to express exogenous proteins, such as calcium sensors or light-sensitive ion channels, that can be used to study neuronal function. In this review, we address these uniquely powerful features of the viruses already in the neuroanatomist’s toolbox, as well as the aspects of their biology that currently limit their utility. Based on the latter, we consider strategies for improving viral tracing methods by reducing toxicity, improving control of transsynaptic spread, and extending the range of species that can be studied.

## Introduction

### The Brain: a Problem of “Organized Complexity”

In 1958, Warren Weaver (Weaver, 1958), a mathematician and former Director for the Natural Sciences of The Rockefeller Foundation, introduced an important distinction between what he called “problems of disorganized complexity” and those of “organized complexity.” The former consist of situations in which a very large number of individual entities might be involved, such as the individual molecules within a container of nitrogen gas, but where the interactions between the entities were helter-skelter; that is, there was no special relationship between any one molecule and any other. The aggregate behavior of such systems could be remarkably well described using statistical methods, one important example being the development of statistical mechanics in the late 19th century by Gibbs, Boltzmann and others. The second class of problems—those of *organized* complexity—were different in the key respect that the particular ways in which the individuals interacted was a critical part of the problem. This may consist of individual humans participating in a market economy, of honeybees within a hive, or of neurons within a brain.

In this last case, it is the connections that define the emergent behavior of the system. Knowing these is arguably as or more important than knowing the precise location of the individual neurons—a possibility that was imaginatively explored in Arnold Zuboff’s “The Story of a Brain,” (Zuboff, 1981) in which his mad-scientist characters create a “spread-brain” where the brain’s individual hemispheres (and, ultimately, individual neurons) could be kept alive in separate nutrient baths, preserving the brain’s owner’s consciousness so long as they were appropriately wired together. Practical (and ethical) issues aside, this idea that if one understood all of the brain’s connections—the so-called “connectome”—one would be a long way towards understanding how it works, continues to drive modern neuroanatomy.

### A Brief, Focused History of Connection Mapping with Non-viral Tracers

In this section, we briefly describe the historical development of non-viral tracers, mainly as it pertains to issues relevant to the viral tracers we discuss below. For readers desiring a more thorough and technical treatment of this topic, we have provided relevant references in Table 1 and further recommend several excellent reviews: for early, transport-based methods, see Cowan and Cuenod (1975) and for more recent methods Lanciego and Wouterlood (2011) and Wouterlood et al. (2014).

**Table 1:** **Overview of non-viral neuroanatomical tracers**.

Family	Examples	Dir	Spd	Fill?	Reference
**Small proteins**	Horseradish peroxidase (HRP) albumin	**R**/A	F	N	Kristensson and Olsson (1971) LaVail and LaVail (1972)
**Inorganic fluorescent molecules**	Fast Blue (FB)	R	M	N	Kuypers et al. (1979)
	Diamidino yellow (DY)				Bentivoglio et al. (1980)
	Fluoro-gold (FG)				Schmued and Fallon (1986)
**Dextrans**	Fluoro-Ruby (FR)	**A**/R	M	N	Glover et al. (1986)
	Biotinylated dextran amine (BDA)	**A**/R		Y	Nance and Burns (1990)
					Veenman et al. (1992)
**Lectins**	Wheat germ agglutinin (WGA; WGA-HRP)	**R**/A	F	N	Schwab et al. (1978)
	*Phaseolus*				Gonatas et al. (1979)
	*vulgaris*-leucoagglutinin (PHA-L)	A		Y	Gerfen and Sawchenko (1984)
**Beads**	Latex microspheres	R	F	N	Katz et al. (1984) Katz and Iarovici (1990)
**Bacterial toxins**	Tetanus	R	F	N	Stoeckel et al. (1977)
	cholera (B fragment)	**R**/A			Schwab and Agid (1979)
					Trojanowski et al. (1981)
**Trophins**	Nerve growth factor (NGF) brain-derived neurotrophic factor (BDNF)	R	F	N	Hendry et al. (1974) Stoeckel and Thoenen (1975)
**Amino acids**	^3^H-leucine	A	F/S	N	Cowan et al. (1972)
	^3^H-proline			Y	Hendrickson (1982)
	biocytin				King et al. (1989)
**Carbocyanine dyes**	DiI	A/R	S	N	Honig and Hume (1986)
	DiO				Honig and Hume (1989)

*Abbreviations: “Dir”, direction; “A”, anterograde; “R”, retrograde (for bi-directionally transported substances, bold-face indicates predominant direction or most common usage); “Spd”, speed; “F”, fast; “S”, slow; “M”, moderate; “Fill?”, Does the tracer produce Golgi-like fills of neuronal cell bodies?; “Y”, yes; “N”, no*.

With Augustus Waller’s (1850) histological description of degenerating axons following section of the glossopharyngeal and hypoglossal nerves of frogs, it became possible to use “Wallerian degeneration” to trace the course of nerve fibers within the brain. This lesion-based method was greatly augmented by the discovery that the degenerating nerve terminals could be labeled with metallic silver (Hoff, 1932; Glees, 1946; Nauta and Gygax, 1951; Fink and Heimer, 1967). In a separate development, the silver-based Golgi method, most famously exploited by Ramón y Cajal (1909), allowed one to visualize entire neurons, including their dendrites and unmyelinated axons, thus permitting a scientist with a superb visual imagination, such as Cajal possessed, to map out the first “circuits” with surprising perspicuity. While the Golgi method produced beautifully labeled neurons whose morphology could thus be studied in detail, it did not permit the reliable demonstration of long-distance anatomical connections; this required the discovery of molecules that would be taken up and actively transported by neurons.

The very fact of Wallerian degeneration made it clear that the distal part of the axon was somehow dependent for its viability on substances supplied by the cell body, and, consequently, that there must exist mechanisms to transport these substances. This deduction was experimentally confirmed by Paul Weiss and colleagues at the University of Chicago (Weiss and Hiscoe, 1948) and not long thereafter exploited to trace connections with the use of radioactively tagged amino acids that were taken up by cell bodies, incorporated into proteins and transported *anterogradely* to axon terminals where they could be rendered visible with autoradiography (Taylor and Weiss, 1965; Lasek et al., 1968). This method, while subject to many limitations (see Cowan and Cuenod, 1975 for details), represented a revolution in neuroanatomy, as it was the first time that connections could be directly demonstrated without having to first inflict damage on the structures of interest. This opened the door to experiments combining connectivity mapping with measurements of neuronal function and development.

The first method that exploited the *retrograde* transport of substances by axons was that demonstrated by the intramuscular injection of proteins, such as, initially, fluorescently labeled albumin (Kristensson, 1970), and then, with greater success, horseradish peroxidase (HRP; Kristensson and Olsson, 1971) which labeled the appropriate pool of motor neurons. HRP was subsequently shown to work in the central nervous system as well (LaVail and LaVail, 1972). It is interesting to note that the early pioneers of these methods were well aware of the powerful cell biological phenomena into which they had tapped. The final sentence of the paper by Kristensson and Olsson (1971) is particularly prescient: “The finding that axons are capable of taking up exogenous proteins and transporting them in a retrograde direction to the nerve cell body may have important implications for the understanding of certain puzzling neurobiological phenomena such as trophic influences of end-organs on the nerve cell body, the signal for chromatolysis after axonal lesions, and how certain toxins and neurovirulent viruses spread from the periphery to the central nervous system.”

The use of HRP provided an important benefit over albumin, because its enzymatic activity could be exploited to produce a visible reaction product when post-mortem sections were incubated in a mixture of hydrogen peroxide and diaminobenzidine (Graham and Karnovsky, 1965), a feature that was further improved upon by the use of other substrates, most notably tetramethyl benzidine (Mesulam, 1982). The latter method could produce a reaction product consisting of birefringent crystals that, when viewed under darkfield illumination, revealed brightly labeled neuronal cell bodies on a black background. Even so, these methods did not completely fill neurons and their dendritic arbors, so while retrogradely labeled cell bodies could be identified, their morphology remained poorly defined (though, see Keefer et al., 1976). It was only later, with the discovery of tracers such as biocytin (King et al., 1989), *Phaseolus vulgaris*-leucoagglutinin (PHA-L; Gerfen and Sawchenko, 1984) and biotinylated dextran amine (BDA; Veenman et al., 1992), that “Golgi-like” fills of projection neurons became possible.

While HRP continued to be a workhorse for neuroanatomists, it lacked the desired degree of sensitivity, because its uptake by neurons at the injection site was relatively inefficient. This was improved dramatically by conjugating HRP to certain plant lectins, especially wheat germ agglutinin (WGA; Gonatas et al., 1979), a breakthrough that took tracing connections from something of a dark art to a reliable technique that even physiologists could use (M. S. Livingstone, personal communication). Motivated by the notion that the improved sensitivity was due to interactions of the lectins with specific cell-surface molecules on neurons, investigators also explored other conjugates, including cholera toxin (Trojanowski et al., 1981, 1982) and tetanus toxin (Stoeckel et al., 1975; Schwab and Agid, 1979), which were known to be retrogradely transported by neurons and which were subsequently shown to selectively interact with specific gangliosides that were enriched on the surfaces of neurons (Stoeckel et al., 1977).

While this brief history omits a number of important tracers, such as the inorganic fluorescent dyes discovered by Kuypers and colleagues (Kuypers et al., 1979; Bentivoglio et al., 1980), we can already begin to see many of the key properties that would make for an ideal tracer. First, it would be selectively and efficiently taken up by neurons, preferably by intact cell bodies or axon terminals and not damaged fibers (i.e., “axons of passage”); second, it could be targeted to a specific class of intracellular motor proteins that would selectively transport it in either the anterograde or the retrograde direction (Dodding and Way, 2011; Maday et al., 2014); third, upon arriving at its destination, it could be readily amplified and rendered visible, providing a Golgi-like fill with minimal need for histological processing; and fourth, it would be available in a variety of different, distinguishable colors so that experiments with multiple tracers could be performed. A fifth property, not directly addressed above, but a property of some classical tracers, e.g., (Schwab et al., 1979), is that it could potentially cross synapses to label second- and, possibly, higher-order neurons in a given pathway.

Tracers exhibiting some of these properties have been discovered over the past several decades by a combination of trial-and-error, educated guesses and luck. However, given the vast time and superior tinkering skills available to Mother Nature, one is not surprised to learn that she has already produced devices with all of these properties, in the form of neurotropic viruses. What’s more, because they carry information encoded by nucleic acids, they can be genetically engineered for specific desired properties (e.g., to travel in anterograde vs. retrograde directions; or to cross or not cross synapses—see “Engineering Viruses to Control Transport” Section) and also be used to introduce foreign genes that can be used to easily detect their presence [e.g., green fluorescent protein, (GFP)] or, more powerfully, to directly monitor or manipulate neuronal function (see “Viruses Engineered to Carry Functional Genes” Section). After a primer on viruses, we will describe some of the modern neuroanatomical strategies permitted by viruses and their genetically altered cousins, ending with a discussion of possible directions for further development of these powerful biological tools.

## A Primer on Viruses for Neuroanatomists

Each gene transfer application calls for a particular set of features in a *vector*, which is defined here as a derivative of a virus used for the delivery of genes. They include: target cell type(s), vector genome size, expression level and duration, vector concentration (titer), DNA or RNA genome, speed of expression after infection, ease of altering the genome and capsid, and safety. One other property of note is whether the virus has an envelope, a lipid bilayer surrounding the capsid, derived from the host plasma membrane. Enveloped viruses have glycoproteins inserted into their envelope, and one can substitute the glycoprotein of one virus for another, to create “pseudotypes,” which can alter the host range of the virus (Hirst and Gotlieb, 1953). Viral vectors commonly in use by neuroscientists, summarized in Table 2, will be discussed in the next section, with a focus on the features that underlie the choices of each vector for application by neuroscientists. However, prior to the discussion of specific properties, it is worth considering the overarching replication potential of a vector, and how this relates to its use by neuroanatomists.

**Table 2:** **Summary of viruses of interest to neuroanatomists**.

Family	Genome	Size	Titer*	Comments/Applications	Dir	Cyto-toxic?	Bio-safety level
**Retroviridae** Gamma-retrovirus, e.g., Moloney Murine Leukemia virus (MMLV), lentivirus, (e.g., HIV)	Positive-strand, single strand RNA that is made into cDNA	9–12 Kb	10^7^–10^9^ cfu/ml	Viral genome integrates into host genome. Use gamma-retrovirus when target cells are dividing and want stable expression; use lentivirus when target cells are not dividing; expression level is typically relatively low	–	N	BSL1–2
**Parvoviridae** Adeno-associated virus (AAV)	Single strand DNA	4.8 Kb	10^12^–10^13^ gc/ml	Do not integrate; work well with neurons for stable expression, small genome can limit use of cell-type-specific enhancer/promoter, or large cDNAs; high titer allows for co-infection with >1 genome to enable combinations of genes	–	N	BSL1
**Adenoviridae** Human adenovirus 5 (Ad5)	Double strand DNA	36 Kb	10^10^–10^12^ vg/ml	Do not integrate; replication-incompetent forms used for rapid and transient expression in neurons; capsids can elicit inflammatory response. CAV is used as a replication-incompetent retrograde tracer	–	N	BSL2
Canine adenovirus (CAV)	Double strand DNA	36 Kb	10^10^–10^12^ vg/ml	As above for Ad5	–	N	BSL2
**Herpesviridae** Alpha-herpesviruses, HSV-1 and Pseudorabies virus (PRV)	Double strand DNA	153 Kb (HSV-1); 144 Kb (PRV)	10^7^–10^9^ cfu/ml	Large and complex genomes, making for more difficult genome engineering; replication-competent and conditional versions are used for transsynaptic tracing	**R**/A	Y	BSL2
				Two versions of replication-incompetent forms, short-term (ST) and long term (LT), are used for gene transfer into neurons; large genome capacity and rapid onset of expression; can achieve co-infection with >1 genome; can go retrograde from injection site	**R**	N	BSL2
**Rhabdoviridae** Rabies virus (RABV), Vesicular stomatitis virus (VSV)	Negative-strand, single strand RNA	12 Kb	10^8^–10^9^ cfu/ml (**Δ**G) 10^10^–10^12^ cfu/ml (wt)	Simple genome makes engineering straightforward; offer good strategy for selection of gene expression level; genome can NOT be recombined with DNA recombinases (Cre and Flp)	**R**/A	Y	BSL1–2
**Alphaviridae** Sindbis virus (SIN) Semliki forest virus (SFV)	Positive-strand, single strand RNA	12 Kb	10^8^–10^9^ cfu/ml	Rapid expression makes it well suited for delivery of transgenes on a short time scale	–	Y	BSL2

*Abbreviations: wt, wild type, i.e., replication-competent, meaning virus can spread, ΔG, G gene is deleted rendering virus unable to spread unless the G gene is supplied* in trans, (i.e., by another genetic element such as AAV), Dir, direction of transsynaptic transmission; “A”, anterograde; “R”, retrograde (for bi-directional transsynaptic transmission, bold-face indicates predominant direction or most common usage).*Titer: The concentration of virion particles and/or infectious units are measured differently for different viruses. While the concentration of infectious units is the most useful for applications, such as cfu/ml (colony forming units/ml) or pfu/ml (plaque forming units/ml), these are not always measured for technical reasons. Alternative methods typically assay viral genome concentration, and different descriptions are used in different publications, e.g., gc/ml (genome copies/ml) or vg/ml (vector genomes/ml). This is important to note as the ratio of particles to infectious particles (p/pfu) can be very high, from 10–10,000. The titers for a virus preparation vary widely due to effects of the genome alterations on virus replication and the preparation method; a range is listed in the Table but there are stocks outside of this range. References: An overview of all of the viruses listed in Table 2 can be found in the following virology textbooks: (Flint, 2009; Knipe and Howley, 2013).

To use a vector as a transsynaptic tracer, it must replicate, that is, produce more infectious virion particles in the initially infected (or “starter”) cell, so that a connected cell can be infected by transmission. A virus that can replicate is referred to as “replication-competent,” and examples include the original forms of pseudorabies virus (PRV; Card and Enquist, 2014) and rabies virus (RABV; Kelly and Strick, 2000). Another type of vector is crippled by deletion of an essential gene, but replication can occur when the missing gene is supplied, *in trans*. These “replication-conditional” vectors have an advantage in that replication can be controlled through the delivery of the missing component. Examples of replication-conditional vectors are the Cre-dependent PRV (DeFalco et al., 2001) and glycoprotein (G)-deleted RABV (Wickersham et al., 2007a). A third category consists of vectors that are unable to replicate at all, or “replication-incompetent” vectors. Some replication-incompetent vectors are useful to neuroanatomists in that they can be used as retrograde tracers; that is, they can be taken up by axonal terminals, whereupon they travel to the cell body and initiate vector gene expression [e.g., herpesvirus (HSV) amplicon vectors, Spaete and Frenkel, 1982]. Replication-incompetent vectors do not spread and thus are not transsynaptic tracers. Some of them have been used for an additional role for neuroanatomy, however, in that they can express genes that aid in the transsynaptic tracing carried out by replication-conditional vectors. For example, a viral G gene can be supplied by a replication-incompetent adeno-associated vector (AAV) to complement a replication-conditional RABV (G-deleted) virus. For some viruses, such as HSV vectors, all of these types of replication styles can be created by different types of genome manipulation (Spaete and Frenkel, 1982; Ugolini et al., 1989; Krisky et al., 1998; Lilley et al., 2001).

### Viruses that do not Cross Synapses

#### Retroviridae

A retrovirus is an enveloped, single-stranded RNA virus. Its most salient feature is that it provides for stable, long-term gene expression by virtue of its ability to stably integrate its DNA genome (created from its RNA genome upon entry into a host cell) into a host cell’s genome, and express virally-encoded genes from a wide range of integration sites (Knipe and Howley, 2013). This ability to integrate distinguishes it from other commonly used vectors, such as AAV. Target cells that are dividing gradually lose viral genomes that are not integrated, due to dilution, and thus any application that requires retention of expression over a number of cell cycles must use a virus that integrates. An example of such an application for neuroscientists might be the introduction of a retrovirus encoding a viral receptor, e.g., avian tumor virus receptor A (TVA), into a progenitor zone early in development. Integration would allow expansion of the number of cells with the integrated TVA gene, so that there would be a large number of TVA-expressing cells at some later point, to facilitate targeted infection using tracing viruses that contain the cognate glycoprotein, EnvA (as discussed in “Use of Monosynaptic and Transsynaptic Tracing *in vivo*” Section).

The two types of retroviruses commonly used as viral vectors are the gamma-retroviruses and lentiviruses. The critical distinction between these two types of retroviruses concerns their ability to enter a host cell’s nucleus in order to integrate the viral DNA. Viral gene expression requires that the genome be integrated, so this step is essential to the use of these viruses as vectors. Gamma-retroviruses are unable to gain entry to the nucleus through an intact nuclear envelope, and thus need a cell to undergo a nuclear envelope breakdown during M phase in order to access a host cell’s genome. In contrast, lentiviral DNA can enter the nucleus without nuclear envelope breakdown, and thus can integrate in a postmitotic cell. This feature makes lentiviruses particularly useful for gene transfer into neurons (Naldini et al., 1996). Both types of retroviruses have been modified to render them unable to replicate after infection, i.e., they are replication-incompetent. Lack of replication makes these vectors safe for use in the laboratory (Schambach et al., 2013), and similarly does not lead to disease in animals, with some rare exceptions due to alterations in the host genome due to viral integration (Hacein-Bey-Abina et al., 2003).

Genome size is an important consideration when choosing a vector for a particular application. Lentiviruses and gamma-retroviruses can package a genome of approximately 10 Kb (Kumar et al., 2001), which allows for expression of more than one gene, as well as use of more than one promoter or regulatory element. In contrast, AAV can only package approximately 4.8 Kb. Retroviruses can express cDNAs from the viral long terminal repeat (LTR) promoter, or they can be engineered to use an internal, non-retroviral promoter, e.g., for tissue specificity (Montiel-Equihua et al., 2012). In the latter case, use of a viral genome rigged to lose the activity of the viral LTR appears to give a greater probability of correct regulation of an internal promoter (Miyoshi et al., 1998; Ginn et al., 2003). However, even with such crippled LTRs, internal promoters are not always properly regulated, and the rules that govern regulation are not known. Retroviral vectors can be engineered to express small-hairpin RNAs (shRNAs) from Pol III promoters (Paddison et al., 2004; Harpavat and Cepko, 2006), and can use multiple promoters in the same genome. For example, a lentivirus was designed to use the Pol III promoters, H1 and U6, to express multiple shRNAs targeting complexin-1 and complexin-2. The same virus used the Pol II promoter, derived from the human ubiquitin-C gene, to express an shRNA-resistant form of complexin-1 (Ahmad et al., 2012).

The choice of the type of retrovirus to use is typically driven by whether the target cell type is mitotic or postmitotic. Gamma-retroviruses have been used to trace lineages in the nervous system, as they will only integrate (and thus express) in mitotic cells, which is exactly what one needs for lineage tracing (Turner and Cepko, 1987). As mentioned previously, lentiviruses are used when one wants to deliver genes to neurons. Both types of retroviruses have been used for gene therapy in humans, due to their stable integration, which provides for long-term gene expression, and again, the choice of which type to use takes into consideration whether the target cell is actively dividing (Wiznerowicz and Trono, 2005; Hacein-Bey-Abina et al., 2014).

Another issue to consider in choosing a viral vector is the percentage of cells at an injection site that need to be infected for the experimental outcome. A limitation of retroviruses is that they do not grow to as high a titer (typically in the range of 10^8^–10^9^ colony forming units per mL, cfu/mL) as AAVs or adenovirus vectors, which can be several log_10_ units higher. The expression level should also be taken into consideration. In part, due to the fact that there is usually only a single viral genome in an infected cell, the level of expression from retroviruses tends to be modest (Wickersham et al., 2007a), though a recently developed lentiviral vector with a tetracycline regulatory element and activator may overcome this problem for some applications (Cetin and Callaway, 2014). Relatively low expression is a disadvantage of using these vectors, as not all neuronal processes are well labeled.

The genome structure of retroviruses is fairly flexible, allowing for the design of vectors using different types of promoters and cargoes. In addition, retroviruses, which have an RNA genome packaged in their capsids, create a DNA copy of themselves after infection, which is integrated into the host genome. This means that the integrated viral genome can be modified by Cre- or Flp-mediated recombination (Gonçalves et al., 2010; Sommer et al., 2010), and allows the use of cellular promoters, neither of which is possible when using RNA viruses. The virion particles also can be modified to suit certain applications. Retrovirus particles have a lipid envelope, and can accept the envelope glycoprotein from other viruses, or pseudotyped with, for example, the avian EnvA glycoprotein that targets the infection to cells that express the avian TVA receptor (Bates et al., 1993; Holland et al., 1998). This approach has been used to target infection for lineage studies (Beier et al., 2011a; Hafler et al., 2012) as well as transsynaptic tracing studies using RABV (Wickersham et al., 2007b) and vesicular stomatitis virus (VSV; Beier et al., 2011b).

#### Parvoviridae—Adeno-Associated Virus (AAV)

AAV is a non-enveloped, single-stranded, small DNA virus of the Parvovirus family (Knipe and Howley, 2013) that has proven effective for gene transfer in the CNS (for a recent review of AAV in the CNS, see Murlidharan et al., 2014). AAV can be used for long-term, stable gene expression in neurons, with little or no toxicity (Kaspar et al., 2002). Its other notable feature is that it grows to very high titers, which can allow the simultaneous infection of cells with more than one AAV vector. There are hundreds of naturally occurring “species” of AAV (Gao et al., 2004), referred to as serotypes, which can be exploited for infection of different cell types. In addition to differences in capsid proteins, the different serotypes have different sequences in their inverted terminal repeats (ITRs), sequences required for replication of the viral genome, as well as differences in the viral replicases that recognize these sequences. The ITRs from serotype 2 have been the most extensively employed in AAV vectors. The ITR serotype is the number that is listed first in the name of an AAV vector, while the capsid type is the second number, e.g., AAV2/8 has the ITRs from AAV 2 and the capsid proteins from AAV 8 (Gao et al., 2005). Serotypes vary in terms of the speed of onset of viral gene expression, with some of the serotypes being particularly slow, needing up to several weeks for the maximum number of cells to show evidence of infection (Vandenberghe et al., 2013). Some of the reasons for this remain unknown, but uncoating and the need to replicate a second strand of DNA (as the virus only packages a single DNA strand) contribute to the lag time. AAV vectors with a genome that is self-complementary, and thus are double-stranded in the capsid, are much faster in terms of onset of expression as they do not need to produce a second DNA strand before initiating gene expression (McCarty et al., 2001; Gray et al., 2011). However, the packaging size of these vectors is very small, about half that of conventional vectors.

Another feature of a serotype to consider is the target cell specificity (e.g., Burger et al., 2004; Cearley and Wolfe, 2006; Taymans et al., 2007; Zincarelli et al., 2008; Aschauer et al., 2013), whether the capsid is transported away from the inoculation site and, if transported, whether in the retrograde (Taymans et al., 2007; Hollis et al., 2008; McFarland et al., 2009; Towne et al., 2010; Masamizu et al., 2011; Aschauer et al., 2013; Castle et al., 2014) or/and anterograde (Castle et al., 2014) directions. AAVs can also access the CNS and PNS from intravascular perfusion, with different efficacies and tropisms depending upon the capsid (Zhang et al., 2011). Toxicity also can vary with the serotype (Howard et al., 2008), or even with a given virus preparation. Toxicity can be due to a high level of expression of the encoded gene, and/or acute inflammation, though this is typically quite low for AAV (McPhee et al., 2006; Zhang et al., 2011; Sondhi et al., 2012), especially relative to adenovirus (Seiler et al., 2007). T-cell mediated toxicity for AAV-infected cells has been seen in humans due to memory T cells (Mingozzi and High, 2013), but has not been reported for rodents. In addition, there may be toxic elements in a virus preparation that do not elicit toxicity through immune system interactions. For example, we have found that different preparations of the same genome in the same capsid type can vary in toxicity, and others have reported differences in toxicity among serotypes for cells cultured from different areas of the CNS (Howard et al., 2008). Acute toxicity in certain preparations may be due to the capsid itself, or to something that is tightly complexed with the capsid, as we have been unable to eliminate toxicity through multiple purification methods. However, dilution of a stock has proven an effective approach to minimizing toxicity.

In general, AAVs have proven to be very useful for long-term, relatively high-level gene expression in the mammalian CNS. In addition, as with retroviruses, their genome is double-stranded DNA after they infect and convert their single stranded packaged genome into a double strand. Their genomes can thus be modified by recombinase technology, with the tightest Cre-dependent vectors using two pairs of loxP sites, called Flip-excision (FLEx) vectors (Schnütgen et al., 2003). Multiple Cre-dependent vectors can be used to simultaneously infect the same cells, but there are some interesting caveats that must be considered when designing such vectors, due to presumed interactions between AAV-encoded loxP sites on two or more vectors. For similar reasons, there appear to be interactions between AAV-encoded loxP sites and genomic loxP sites (Saunders et al., 2012). Cre-dependent AAV vectors have been exploited for transsynaptic tracing protocols and for targeting optogenetic protein expression to specific types of neurons that express Cre (Atasoy et al., 2008; see also “Viruses Engineered to Carry Functional Genes” Section). The popularity of AAV vectors with neuroscientists also derives from the fact that they are straightforward to engineer, and there are multiple companies and core facilities that make virus constructs as well as virus preparations.

AAVs can be produced as high titer stocks [10^12^–10^13^ genome copies per mL (gc/mL) for AAV]. Note that AAV titers are usually given in gc/mL, and retroviral titers in infectious units (e.g., cfu/mL). AAV stocks using different capsid types, and different preparations of a given capsid type (Auricchio et al., 2001), can vary in their ratio of capsids to infectious capsids, also referred to as the particle to infectious particle ratio (p/pfu), or infectivity. These ratios range from 10–1000:1 (Auricchio et al., 2001; Vandenberghe, personal communication) and will affect the percentage of cells infected at an injection site. Due to the fact that it is not always trivial to measure infectious particle activity for AAVs, the p/pfu ratio is usually not measured but is one reason why stocks vary in their infectivity. The variables of toxicity and infectivity argue for pilot experiments with each new stock before investing a great deal of time in its use.

Although the naturally occurring AAVs integrate their genome into the host chromosome, at a specific locus on human chromosome 19, the deletion of the rep genes from the vector form of AAV has led to the loss of such targeted integration (McCarty et al., 2004). Some random integration occurs at a low level, but it is not clear if such integrated genomes express viral genes. Most AAV genomes exist as non-integrated episomes, either as single viral genomes or as concatamers (Yang et al., 1999; Nakai et al., 2000). The reason(s) that these genomes remain stably associated with the host cell and persist in expression has not been determined. The lack of consistent integration makes AAV vectors a poor choice if the target cells are dividing and retention of viral gene expression through several cell cycles is required. As noted above, in such cases, retroviruses are a better choice.

The natural promoters of AAV have been replaced with promoters from other viruses, e.g., cytomegalovirus (CMV; Boshart et al., 1985), or host cells, e.g., EF1α (Fitzsimons et al., 2002). Other regulatory elements that can be included are introns, to provide for splicing, polyA sites, and a regulatory element known as the woodchuck post-transcriptional regulatory element (WPRE), which increases the cytoplasmic level of virus-encoded mRNAs (Donello et al., 1998; Zufferey et al., 1999). As with all viral vectors, the exact sequences and their arrangement in the vector can have effects on the level of expression as well as the specificity of expression. As an example, we have found that different intron sequences can have a major effect on the level of expression in the retinal pigmented epithelium (Xiong et al., Unpublished observations). Due to these variables, one should assay for expression of an easily scored gene, such as GFP, using a vector that has all of the regulatory elements and the capsid serotype that one wishes to use in future experiments. In addition, such tests should be performed on animals that are of the age that will be used for future experiments, as we have found that the age of the tissue at the time of inoculation can affect the types of cells infected (Xiong and Cepko, 2014). One can obtain a series of relatively inexpensive, small aliquots of different serotypes from companies to test for the optimum vector characteristics.

The major drawback of AAV vectors is their limited packaging capacity. AAVs can only incorporate about 4.8 Kb of DNA (Dong et al., 2010), which limits the expression to one or two small genes, and also limits the use of different promoter/enhancer elements. There have been several approaches to overcome this limitation. For example, there are trans-splicing vectors where two genomes with different exons of a large gene are co-injected, and result is expression of a large protein, but only if there is a high rate of co-infection (Wu et al., 2010). However, generally if a larger genome is desired, another vector, such as an adenovirus or lentivirus, should be considered.

#### Adenoviridae—Adenovirus

An adenovirus is a non-enveloped, double-stranded DNA virus, with a larger genome and capsid than AAV, but not as large as those of the HSVs (Knipe and Howley, 2013). Human adenovirus type 5 (Ad5) has been developed for use for gene therapy and most commonly used adenovirus vectors are derived from Ad5 (Gonçalves and de Vries, 2006). The newest generation of adenoviruses (helper-dependent adenovirus or HDV) have had all of their genes removed, both to make the virus less susceptible to immune system modulation and to create space for transcriptional regulatory elements and large or multiple genes (Parks et al., 1996; Ehrhardt and Kay, 2005). These HDVs have a capacity of 35–36 Kb. Adenovirus vectors are not quite as straightforward to genetically engineer or produce (Palmer and Ng, 2003) relative to retroviruses and AAVs. In addition, they remain more pro-inflammatory than retroviruses or AAV (Seiler et al., 2007). As with AAVs, they do not integrate and thus are not used for applications where the target cells are mitotic. Also, as with AAVs, they can be grown to high titer, but unlike AAVs, they express at a high level within a few days of infection (Akli et al., 1993). Adenovirus vectors have been directly compared to AAVs and lentiviruses for gene transfer in the CNS, and have not fared well in these comparisons when the time course has gone beyond a few days (e.g., Doherty et al., 2011). Still, transient and immediate expression of a gene, such as Cre, using adenovirus vectors can be useful and high titer stocks that express Cre can be purchased from several vendors.

The canine adenoviruses (CAV) are employed for their potent retrograde transport capabilities (Kremer et al., 2000; Soudais et al., 2001; Kissa et al., 2002; Peltékian et al., 2002; Hnasko et al., 2006). In addition, these vectors have been used to express Cre, and a few other genes, e.g., GFP (Kremer et al., 2000). Cre has been used to turn on the expression of floxed endogenous genes (Hnasko et al., 2006), including floxed genes used to enable monosynaptic tracing by RABV (Pivetta et al., 2014). These vectors may become increasingly useful when a DNA virus is required, as the packaging limit of AAVs often limits the experiments that one can do with them. As adenoviruses have double-stranded DNA genomes, they are amenable to Cre or Flp-mediated recombination.

#### Alphaviridae—Semliki Forest Virus (SFV) and Sindbis Virus (SIN)

The alphaviruses, semliki forest virus (SFV) and sindbis virus (SIN), are enveloped, plus-strand single-stranded RNA viruses. They can be engineered to be replication-incompetent, and typically grow to as high a titer as retroviruses (in the range of 10^8^–10^9^ cfu/mL). However, unlike retroviruses, multiple viral genes are still expressed from these vectors, resulting in rapid gene expression, but also rapid toxicity.

They have been used in cases where this rapid, high expression is desirable (Bredenbeek et al., 1993; de Hoop et al., 1994; Gwag et al., 1998; Ehrengruber et al., 1999). For example, they have been used for robust labeling of a small number of axons of neurons near the injection site in the brain (Ghosh et al., 2011; Kuramoto et al., 2015), as well as for delivering genes to neurons (including a fluorescent tag for visualization) for single-cell electrophysiological experiments (Kopec et al., 2007; Malinow et al., 2010), among others. However, rapid toxicity limits the use of these vectors. The fast expression and toxicity of these alphaviruses make them similar to VSV, though the transgene expression from VSV is even quicker, occurring within hours (van den Pol et al., 2009; Beier et al., 2011b, 2013b), as discussed further below.

### Viruses that Cross Synapses

#### Herpesviridae—HSV and PRV

HSVs are large, enveloped, double-stranded DNA viruses which are classified into three subfamilies (Davison, 2010). PRV and HSV-1 are members of the subfamily, alphaherpesviruses, and are those used for circuitry tracing. Another subfamily is the betaherpesviruses, which includes CMV, one strain of which is the source of the popular human CMV immediate early enhancer/promoter used extensively in expression vectors (Boshart et al., 1985). The herpesviruses were the first to be extensively employed to trace neuronal circuitry. Though some of the early reports of herpesviruses traveling along nerves were made in the 1920s (Goodpasture and Teague, 1923), the first applications of this type of virus for tracing did not begin until the late 1980s (Ugolini et al., 1989). In these early studies, the virus was injected into peripheral locations, and transmission to specific areas in the brain known to project to the spinal cord was observed. This permitted the mapping of brain projections onto these peripheral sites. Later studies also examined the transmission patterns of different strains of HSVs, such as PRV, in the brain (Card et al., 1990; Strack and Loewy, 1990). All of these initial studies used viruses that were replication-competent and thus could spread across many synapses. The fact that HSV’s can spread raises a question of safety for laboratory workers. As PRV does not infect primates from peripheral sites of infection, this is not a major concern, though the virus can replicate, and kill neurons, when cells of the CNS are exposed to the virus, by direct injection (Hurst, 1933) or nasal instillation (Baskerville and Lloyd, 1977). HSV-1, in contrast, is a natural pathogen of humans, giving rise to genital and oral lesions (Lafferty et al., 2000). In addition, these viruses are toxic to cells, as is true with the other viral transsynaptic tracers.

Specific strains of the virus are able to transmit only in the retrograde direction, as in the case of the PRV Bartha strain (Card et al., 1992; Levine et al., 1994; Moore et al., 1995). Other strains, such as the HSV-1 strain H129 (Zemanick et al., 1991; Sun et al., 1996), appear to transmit transsynaptically only in the anterograde direction. The retrograde specificity of the Bartha strain is likely due to a large deletion in the viral genome, which eliminates the expression of three viral genes (Lomniczi et al., 1987), while the anterograde specificity of the HSV H129 is not understood (Szpara et al., 2010). The mechanisms of axonal transport, both retrograde and anterograde, have been studied, and while there is still much to be learned, the process is better understood than for the rhabdoviruses, RABV and VSV. However, for a good review of how difficult it is to clearly define these mechanisms, see Kratchmarov et al. (2012).

**Figure 1:**
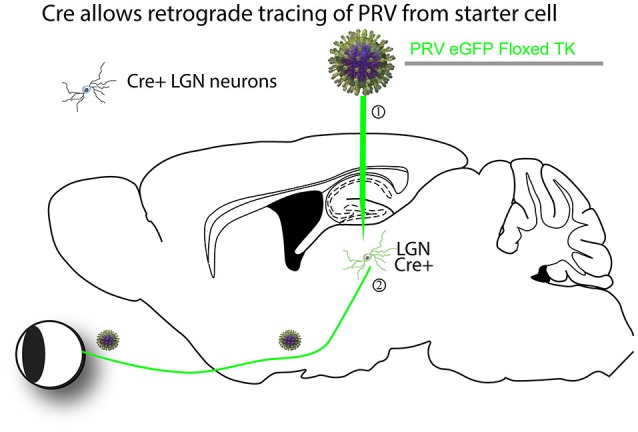
**An example of a Cre-dependent tracing strategy.** The first Cre-dependent tracing virus was a PRV that expressed a gene essential for virus replication in neurons, thymidine kinase (TK), in a Cre-dependent manner (DeFalco et al., 2001). A theoretical example is as follows. (1) The virus is first injected into a region of interest with cells expressing Cre. (2) Cre recombines the PRV genome so that it can express GFP and TK. The virus can then transsynaptically move from the starter cells to input neurons, such as retinal ganglion cells (RGCs) in the retina in this theoretical example. The virus can continue to transmit polysynaptically, as once Cre recombined the viral genome, it is permanently activated for transmission.

HSVs are DNA viruses, and thus their genomes can be engineered using standard recombinant DNA methods. However, as these viral genomes are very large, about 150 Kb, and have many regulatory elements and genes, even minor genome alterations can cripple the virus’s ability to replicate. Since at least one cycle of replication is required for a virus to transmit across a synapse, any modification that affects viral fitness can greatly reduce the utility of a construct. Some modifications have been made to enable the use of these viruses as tracers. Fluorescent protein genes have been successfully inserted, and there are versions which include the Brainbow or rainbow cassettes of fluorescent proteins (Boldogkoi et al., 2009; Kobiler et al., 2010). In addition, there is a version that can be used as a calcium sensor, and through the use of both GFP and DsRed, there is a version which can be used to indicate the timing of arrival of the virus in a circuit, as well as the health of infected cells (Boldogkoi et al., 2009). PRV and H129 also have been rendered dependent upon Cre expression for replication, allowing one to use Cre expression patterns in transgenic mice to dictate tracing of a particular circuit (DeFalco et al., 2001; Lo and Anderson, 2011; Figure 1), or to express a variety of transgenes which can aid in circuit mapping (Boldogkoi et al., 2009; Kobiler et al., 2010). As mentioned above, PRV and HSV-1 are toxic to cells, and have been suggested to have the ability to alter neuronal connectivity (McCarthy et al., 2009). However, a variant of PRV was recently generated that lacks the master transcriptional regulator of endogenous PRV genes, and can therefore express transgenes without replicating or apparent toxicity (Oyibo et al., 2014), auguring an expanded use of these vectors. In terms of speed, PRV has been seen to label infected tissue culture cells in 6 h (Kobiler et al., 2010), and initially infected neurons *in vivo* in just over 1 day post-infection, while the first instances of spread *in vivo* were seen at around 2 days post-infection (e.g., Card et al., 1990). As with all transsynaptic tracer viruses, the kinetics of expression are slower in variants that reduce the fitness of the virus. In addition, the kinetics of labeling of circuits are determined by such variables as the distance of the injection site to the cell bodies of the initial target cells, and the distances from the initial sites of replication to the cell bodies of the secondarily infected cells. Titers of these HSV vectors are typically 10^7^–10^9^ cfu/ml, and the p/pfu is variable and can be up to 40,000 (Flint, 2000) perhaps due to the fact that the virion particle is large and complex, leading to failures in assembly and/or uncoating.

In addition to its utility as a transsynaptic tracing vector, HSV-1 has been engineered to be a replication-incompetent retrograde tracer, and to be an effective vector for other types of gene transfer into neurons (Spaete and Frenkel, 1982; Krisky et al., 1998; Samaniego et al., 1998; Lilley et al., 2001). These replication-incompetent vectors are not toxic to cells, and can be made into forms that express for different lengths of time, including several months. Given their large size, and certain aspects of vector design, they can be engineered to express at high levels, to express multiple genes, and to use large and/or multiple regulatory elements. They are somewhat more difficult to produce than some of the other vectors discussed here, but there are core facilities that can produce them.

#### Rhabdoviridae—RABV and VSV

Rhabdoviruses are enveloped, single-stranded RNA viruses, with two members, RABV and VSV, serving as transsynaptic tracers. RABV and VSV each encode five genes, with a genome of approximately 12 Kb. Their capsids are flexible enough to allow packaging of genomes both significantly smaller (Epstein et al., 1980; Timm et al., 2014), or larger (McGettigan et al., 2003), than the native genome. The genes of RABV and VSV are expressed at high levels, permitting robust gene expression and visualization shortly after infection. RABV and VSV have a simple genome organization optimized for the levels of expression of each gene, with a gradient of expression from the 3′-most gene (highest) to the 5′-most (lowest). RABV is typically engineered to encode non-viral genes at the G locus, which is in the fourth position. This means that the expression level of such a gene (Osakada et al., 2011) is less rapid and robust than that of a gene in the first (most 3′) position (van den Pol et al., 2009; Beier et al., 2011b, 2013b; Osakada et al., 2011). However, vectors can be made in which the fluorophore is placed at the 3′-most end, which results in more robust expression (Wickersham et al., 2013).

Due to fact that the genome is RNA, and rhabdoviruses do not have a DNA phase in their lifecycle, it has been challenging to produce infectious virus from engineered plasmids that represent the genomes (Whelan et al., 1995; Mebatsion et al., 1996). Nonetheless, methods have been worked out, and after a virus preparation is made from engineered plasmids, they are then straightforward to propagate by simply infecting host cells *in vitro* (Osakada et al., 2011). It is worth noting that the lack of a DNA phase in their replication also means that one cannot use DNA recombinases, such as Cre, to recombine their genomes. A summary of the unique features of RABV and VSV as transsynaptic tracers is given below.

#### RABV

RABV, like HSV, can infect neurons from the axon terminals and be retrogradely trafficked to the cell body, as well as transmit among neurons transsynaptically in the retrograde direction (Astic et al., 1993; Ugolini, 1995; Kelly and Strick, 2000). Its use has been more limited than that of HSV, however, due to the biosafety concerns of using RABV, as it is often lethal upon human infection. However, it has been very useful for retrograde transsynaptic tracing in non-human primates (Nassi and Callaway, 2006; Nassi et al., 2006; Rathelot and Strick, 2006, 2009).

The first studies linking RABV to transmission from synaptic junctions occurred in the 1970s and 1980s (Iwasaki et al., 1975; Charlton and Casey, 1979; Tsiang et al., 1983). This was followed by studies in which the time-course of RABV infection was monitored in order to construct circuit diagrams. In one such early study, the tongue muscle was injected with RABV followed by survival times of 1–4 days in different animals (Ugolini, 1995). As expected, after a single day, only first-order hypoglossal motor neurons were labeled. Between 2 and 3 days post-injection, second-order neurons in various brainstem nuclei were identified, while 4-day survival times resulted in the labeling of putatively third-order neurons, including forebrain nuclei. This approach permitted the identification of a chain of connections based on the timing of transsynaptic transmission of RABV. Similar studies using transsynaptic transport of RABV (CVS-11 strain) have revealed connectivity in the monkey visual system (Kelly and Strick, 2000; Nassi and Callaway, 2006; Nassi et al., 2006).

However, the biosafety concerns inherent in using a replication-competent RABV made the use of this virus restricted to a small number of labs that were proficient in its handling and use. A major advance occurred in 2000, when RABV was engineered to become replication-conditional by removal of the G gene from the genome (Etessami et al., 2000). This virus could be used in the laboratory to robustly label neurons projecting to a defined site (Nassi and Callaway, 2007; Wickersham et al., 2007a). In addition, because the virus could not spread, it became more accessible to neurobiology labs who were less familiar with viruses.

The next major advance was the use of this replication-conditional virus for *monosynaptic* retrograde transmission (Wickersham et al., 2007b). This technology permitted, for the first time, unambiguous identification of synaptic inputs onto defined neuronal types. It was also the first virus whose transsynaptic specificity was physiologically verified (Wickersham et al., 2007b). This has led to the widespread use of these vectors to trace circuits in the rodent (discussed further in “Engineering Viruses to Control Transport” Section).

As with HSVs, RABV is toxic to cells. While less rapidly cytotoxic than other transsynaptic vectors such as VSV (van den Pol et al., 2009; Beier et al., 2011b), and HSV-1 and PRV (McCarthy et al., 2009), physiological analyses on RABV-infected neurons should be performed with caution. Also, while many studies have reported the neuronal specificity of RABV transsynaptic tracing, others have reported that glial cells are occasionally observed from viral infection, just as with PRV (Viney et al., 2007; Marshel et al., 2010) and VSV (Beier et al., 2011b). Therefore, viral transmission is not always exclusively limited to traditional synapses.

#### VSV

The activity of VSV in the CNS was not studied as early as the other transsynaptic tracers, though a few studies reported replication in the CNS (Lundh, 1990; Plakhov et al., 1995; van den Pol et al., 2002, 2009). The first study to modify the VSV genome and examine its transmission patterns showed that the virus could indeed cross synapses and label neurons (Beier et al., 2011b). Importantly, the direction of transmission was shown to be dictated by the nature of the glycoprotein. VSV with its own G showed an *anterograde* transsynaptic transmission pattern, while VSV encoding the RABV G showed a *retrograde* transsynaptic pattern (Beier et al., 2011b).

As VSV is non-pathogenic in nature, one can safely use VSV as a polysynaptic tracer, that is, in its replication-competent form. Infection of a peripheral tissue leads to a rapid shut-down of VSV replication, via the innate immune system (Junt et al., 2007). The lack of escape from this immune response renders VSV safe for laboratory use, which is one of the reasons why VSV has been well studied by virologists and cell biologists. However, direct injection of wild type VSV into the CNS can lead to rapid spread in the brain, which can kill mice very quickly, particularly young mice (Sabin and Olitsky, 1937). The recombinant forms of VSV that encode fluorescent proteins and other viral glycoproteins, such as RABV-G, are attenuated and spread in the CNS more slowly than the wild type (Roberts et al., 1998; Beier et al., 2011b). Other genome modifications have been made to create a monosynaptic tracer (Beier et al., 2011b), following the strategy used for monosynaptic RABV (Wickersham et al., 2007b). When the glycoprotein from the lymphocytic choriomeningitis virus (LCMV-G) was supplied *in trans* in hippocampal organotypic slices, VSV was observed to transmit between neurons in an anterograde direction. This was confirmed electrophysiologically, by optically stimulating the starter cell and recording from the post-synaptic neuron (Beier et al., 2011b). VSV is currently being developed for use *in vivo*, for monosynaptic and either anterograde or retrograde transmission.

## Engineering Viruses to Control Transport

### Monosynaptic and Restricted Transsynaptic Tracing Systems Offer Advantages in Interpretation

Viruses that can cross multiple synapses have proven highly valuable in constructing connectivity maps in various systems. The general strategy has been to use progressively longer survival times after injection to label progressively higher order neurons, as discussed for RABV in the previous section (e.g., Ugolini, 1995).

While such studies have been very informative, they suffer from two major limitations. One is that the virus infects neurons indiscriminately at the injection site, making it impossible to distinguish differences in inputs to different cell types that are intermingled at one location. A second limitation is using survival time to establish the order of connectivity. Variability in viral replication, intracellular transport and neuronal geometry may create timing differences in labeling that do not strictly reflect synaptic order in a chain of connected neurons. For example, a weak, distal input to a large pyramidal neuron may be transsynaptically labeled considerably later than a strong, proximal input, thus leading to confusion between distal, or weak, n^th^ order connections and proximal n + 1^th^ order connections.

The first report of a cell-type specific virus was published in 2001 (DeFalco et al., 2001). PRV was modified to make the expression of a gene necessary for viral replication, thymidine kinase (TK), Cre-dependent so that tracing was only initiated from cell types expressing Cre (Figure 1). However, as the virus could spread across multiple synapses after activation by Cre, it was uncertain whether the novel connections identified represented direct monosynaptic inputs to starter neurons.

**Figure 2:**
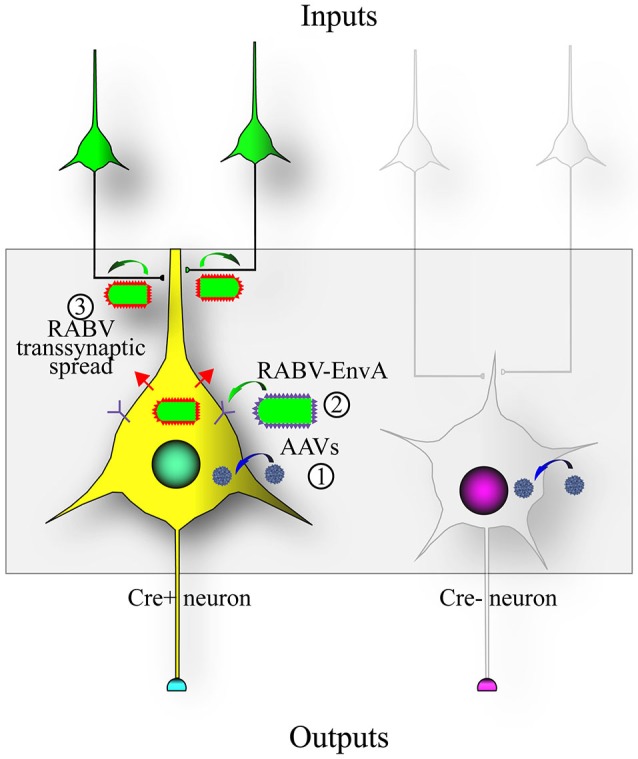
**Design of the RABV monosynaptic technique.** Expression of the avian tumor virus receptor A (TVA) dictates the types of cells that can be infected by a transsynaptic tracing virus that has a glycoprotein with the extracellular domain of a type A avian retrovirus (EnvA). A chimeric protein with the RABV-G intracellular domain fused to the EnvA extracellular domain (aka ASLV-A/RABV-G fusion, or A/G) is often used in place of the native EnvA. (1) The TVA and RABV-G genes are encoded as Cre-dependent genes in adeno-associated virus (AAV) vectors (blue virions) that are injected into an area with Cre-expressing neurons; (2) After Cre-recombination of the AAV encoded genes, a RABV pseudotyped with EnvA (EnvA shown as purple spikes on the virion surface) can infect the TVA-expressing cells; and (3) transsynaptic transmission of the RABV to synaptically-connected input neurons can occur due to the expression of the RABV-G gene (RABV-G shown as red spikes on the virion surface) in the Cre-expressing cells. This method enables RABV to transmit from an initially infected (“starter”) cell to a directly connected presynaptic cell, but no further.

To address this issue, RABV was genetically modified to allow it to specifically infect desired cell types, as well as to spread only to monosynaptically connected inputs (Wickersham et al., 2007b). A schematic of this process is shown in Figure 2. Cell type specificity was obtained by expressing the viral receptor in cell types of choice, and monosynaptic restriction of transsynaptic spread was obtained by deleting the G gene from the viral genome, and resupplying it *in trans* specifically in cells also expressing the viral receptor. The investigators initially delivered these genes to cortical slice cultures by biolistic transfection, and subsequently applied the cell-type specific RABV to these cultures. They observed that: (1) RABV infection was specific to defined cell types; (2) the RABV G was necessary and sufficient for transsynaptic spread; and (3) they verified electrophysiologically that these neurons were, in 9 of 11 cases, synaptic partners.

### Use of Monosynaptic and Transsynaptic Tracing *In Vivo*

**Figure 3:**
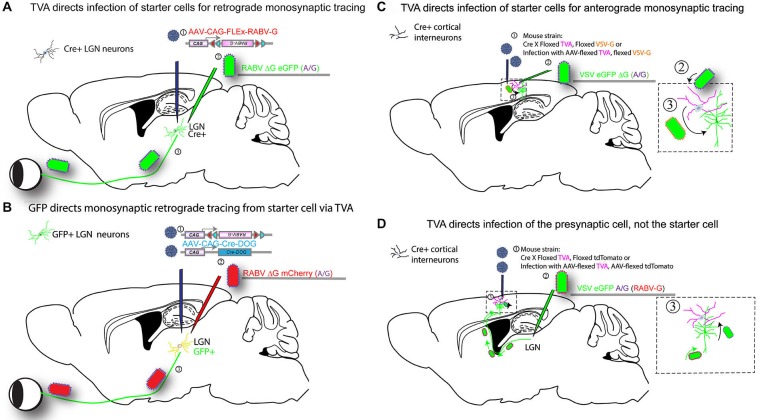
**Infection of TVA-expressing cells determines specificity of tracing.** Expression of TVA can be arranged using transgenic mouse strains, by crossing a strain expressing Cre in a neuronal cell type of interest to a strain with a Cre-dependent TVA. In addition, one can include in this cross a Cre-dependent G protein (e.g., RABV-G) to enable the tracing virus to transmit, and/or a Cre-dependent fluorescent protein to label the cells with Cre history, i.e., the TVA-expressing cells. AAV vectors encoding such Cre-dependent genes can be used as an alternative to transgenic mouse strains (as shown in Figure 2). Four versions of tracing experiments using TVA are shown. **(A)** A transgenic mouse strain that expresses Cre, in e.g., in LGN neurons, will enable identification of the presynaptic partners of the Cre-expression neurons. In this example, the Cre strain is crossed to a strain with a Cre-dependent TVA gene (Beier et al., 2011a), (or, alternatively, an AAV is delivered in step 2 with the Cre-dependent TVA): (1) AAV encoding a Cre-dependent (FLEx) RABV-G is injected into the LGN of mice crossed to have TVA in LGN neurons that are postsynaptic to RGCs, approximately 2 weeks prior to the initiation of tracing. The AAV will uncoat, make a second DNA strand, then initiate expression of the RABV-G protein only in cells that have Cre. Depending upon the serotype of AAV, this process can take over 1 week; (2) A second injection is then made into the same area of the LGN with a tracer virus, RABV ΔG GFP (EnvA or A/G). RABV ΔG GFP will only infect cells with TVA, i.e., those with Cre; (3) When RABV ΔG GFP infects a cell that was previously infected with AAV, and which expresses Cre, RABV ΔG GFP will be transmitted to presynaptic RGCs. Infected RGCs will become GFP+, but will not be able to transmit the virus any further due to the lack of the G gene in the virus and in the RGCs. **(B)** GFP expression determines tracing specificity. The same strategy outlined in **(A)** can be used with a transgenic mouse line that expresses GFP in the LGN: (1) Infection of the LGN with two AAVs encoding Cre-DOG (Cre-dependent upon GFP) enables the GFP-expressing cells to become Cre-expressing cells (Tang et al., in press). Co-infection with an AAV with a Cre-dependent RABV-G and TVA allows the GFP-expressing cells to express RABV-G and TVA; (2) Infection with RABV ΔG mCherry (EnvA or A/G); and (3) transmission from the starter cells to presynaptic cells then proceeds as in scheme shown in **(A). (C)** TVA determines the starter cell type for anterograde monosynaptic tracing. The strategy outlined in A is carried out for anterograde tracing: (1) A mouse cross or AAV are used to determine the TVA-expressing starter cell type. The direction of transsynaptic transmission is set by the choice of G gene, in this case, VSV-G for anterograde tracing. If AAV is used, infection into the cortex is done 2 weeks prior to the initiation of a tracing experiment; (2) Infection of TVA-expressing cells by injection of VSV ΔG GFP (A/G) into the cortex; (3) VSV will replicate in the TVA-expressing cells and if VSV-G is present, will allow transmission to postsynaptic cells. VSV ΔG GFP will not spread from these postsynaptic cells due to the absence of a G gene in the virus and the postsynaptic cells. **(D)** TVA determines the specificity of retrograde infection from starter cells defined by projection site: (1) TVA expression is initiated in cells as shown in **(A)**, but the Cre-dependent RABV-G gene is not used. Instead, one may use a Cre-dependent tdTomato allele to enable the identification of cells with a history of Cre expression (but this is optional). If AAV is used, infection into the LGN is done 2 weeks prior to the initiation of a tracing experiment; (2) A VSV encoding the A/G fusion protein in the genome, and RABV-G protein on the virion surface, is injected, here into the LGN. The RABV-G on the virion surface allows the VSV to travel retrogradely from the LGN injection site to neurons that project into the LGN, e.g., layer 6 pyramidal neurons in V1; and (3) VSV will replicate in the pyramidal neurons and transsynaptically transmit specifically to presynaptic TVA-expressing cells in the cortex. Transmission out of these TVA-expressing cells will only occur to other presynaptic cells that express TVA.

Since the initial demonstration in organotypic slices, the monosynaptic tracing technique has been applied *in vivo* in numerous studies (Figure 3). In the first experiments, investigators used mice expressing Cre in specific cell populations and injected Cre-conditional (FLEx) viruses into the target region (Figure 3A; Wall et al., 2010). In this case, the Cre mice were crossed to a mouse line conditionally expressing TVA, making only the RABV-G necessary to be delivered virally. Other groups have delivered both TVA and RABV-G virally into Cre populations, using either a single virus (Haubensak et al., 2010; Wall et al., 2013) or multiple viruses (Watabe-Uchida et al., 2012; Miyamichi et al., 2013). Another strategy used the tet system, making the expression of tTA2 Cre-dependent, and the AAV expressing TVA and RABV-G used the TRE promoter (Miyamichi et al., 2011). A recent innovation will allow the use of GFP to direct expression of Cre-dependent genes. This system uses AAV encoding Cre-DOG (Cre-*D*ependent *O*n *G*FP), and AAV FLEx vectors, e.g., for TVA and RABV-G (Tang et al., in press; Figure 3B). This is similar to the GFP-dependent transcription system of Tang et al. (2013). It expands the use of transgenic GFP lines for viral tracing through, for example, the selection of GFP+ cells as starter cells. In all of these cases, at least one component is delivered virally (or through the use of Cre and floxed TVA in transgenic mice) in order to spatially restrict the neurons to be infected by RABV.

One additional method that uses TVA and RABV-G for tracing specificity employed a strategy that is the opposite of that outlined above. Rather than use TVA to direct the infection of the starter cells, TVA was used to direct infection of cells presynaptic to the starter cells (Figure 3D; Beier et al., 2013a). A VSV vector with the EnvA fusion protein, ASLV-A/RABV-G (A/G), in the genome was grown *in vitro* with the RABV-G protein supplied *in trans*. This created virions with both the A/G and RABV-G glycoproteins on the virion surface. Infection with this preparation allowed retrograde labeling of cells that projected to the injection site by virtue of the RABV-G on the virion surface. Replication of the virus in the starter cells then led to production of virions with the A/G glycoprotein on the surface, as this was the G encoded by the viral genome. Such virions could only infect presynaptic TVA-expressing cells. In this way, one can interrogate the postsynaptic partners of the TVA-expressing cells, as was done to identify retinal ganglion cells postsynaptic to starburst amacrine cells in the retina (Beier et al., 2013a).

While most studies have used the EnvA-TVA strategy to direct viral infection to specific cell types, other possibilities exist. For example, one method employed a viral receptor-ligand bridge protein. In this strategy, TVB was conjugated to neuregulin, which, when injected *in vivo*, binds to the neuregulin receptor, Erb4 (Choi et al., 2010). This was done in order to make cells with Erb4 on the surface also have TVB on the surface, without requiring these cells to carry the gene expressing TVB. RABV pseudotyped with EnvB, a viral glycoprotein that infects cells with TVB, could then specifically infect Erb4-expressing cells.

In addition to delivery of TVA and RABV-G by AAVs, other studies have provided these genes by single cell electroporation *in vivo* (Marshel et al., 2010; Nguyen et al., 2012). This technique has the advantage of allowing for the physiological characterization of the neurons that are later used for input tracing *before* they are infected with RABV and potentially physiologically compromised by the virus. It also has the advantage of allowing the investigator to conclude that all virally labeled inputs make direct synaptic connections onto a single, identified cell.

The monosynaptic restriction of transsynaptic tracing does not appear to be limited to tracing only in the retrograde direction. Monosynaptic anterograde tracing was first achieved in slice cultures using VSV that expressed the glycoprotein of either LCMV or of VSV itself (Beier et al., 2011b). *In vivo* applications using VSV and VSV-G are currently being developed (Figure 3C). A recent report also suggested that RABV itself may display anterograde transsynaptic transmission in certain systems (Zampieri et al., 2014). Further studies into the mechanisms of viral transsynaptic transmission should shed light on the mechanisms of directional viral transsynaptic transmission, allowing for more precise direction-specific investigation of connectivity.

#### Use of RABV as an Unbiased Screening Method for Identifying Direct, Monosynaptic Inputs

One of the major advantages of the monosynaptic RABV technique is the ability to examine direct synaptic inputs to the targeted neuronal populations without a presupposition about their identity or strength. The advantages of using the monosynaptic virus for this purpose are that it does not label axons of passage, which can be problematic when using some classical tracers, and that its high level of amplification at the cell body allows it to strongly label even weakly connected inputs. For example, this approach was used to determine the inputs to AgRP-Cre expressing neurons in the ventral hypothalamus that are critical for the control of hunger (Krashes et al., 2014). The major inputs were the dorsal medial hypothalamus (DMH, 26% of inputs), and the paraventricular hypothalamus (PVH, 18% of inputs). As the monosynaptic method suggested that these are direct inputs, the investigators then used channelrhodopsin-assisted circuit mapping techniques to show that the strength of these glutamatergic afferents to the AgRP neurons was stronger from the PVH than the DMH, and that these connections subsequently were important for the control of feeding behavior. Another study used the virus to suggest a potential feedforward circuit in the PVH (Betley et al., 2013).

#### Monosynaptic Input Tracing used to Map Circuit Organization

In addition to identifying the presence or absence of connections, RABV can be used to elucidate the topology of neuronal circuits. For example, in the olfactory circuitry in the mouse, it was known that each glomerulus in the olfactory bulb receives projections from one type of olfactory receptor neuron, but it was unknown how the olfactory map was represented in downstream regions, such as in the anterior olfactory nucleus (AON), or in the piriform cortex. RABV was injected into downstream targets of the olfactory bulb projection neurons, the mitral and tufted cells. It was found that the olfactory bulb-AON projection maintained the dorsal-ventral topography present in the olfactory bulb, that the projection to the amygdala was dorsally biased, and that the olfactory cortex appeared to get input from a random assortment of mitral/tufted cells. Thus RABV tracing was able to identify that each of the three different efferent projection sites appeared to have a different representation of the olfactory map than the one present in the olfactory bulb, a finding supported by other studies (Stettler and Axel, 2009; Choi et al., 2011; Sosulski et al., 2011).

These represent only a few examples of the many studies that have used RABV to map connectivity in different circuits throughout the central nervous system (Yonehara et al., 2011; Sun et al., 2014) as well as the spinal cord (Stepien et al., 2010; Tripodi et al., 2011; Esposito et al., 2014).

#### Use of Monosynaptic Input Tracing to Identify Input Specificity of Different Cell Populations

The monosynaptic RABV technique has also been used to quantitatively compare inputs to two or more different cell populations. One study used the virus to compare the inputs to the two major dopaminergic cell populations in the ventral midbrain, the ventral tegmental area (VTA) and substantia nigra pars compacta (SNc; Watabe-Uchida et al., 2012). The authors restricted infection to dopamine neurons using a Cre mouse line (DAT-Cre) and used injections of viruses to target the two anatomical locations. They then counted all of the monosynaptic inputs to each of these regions throughout the entire brain. The first observation was that each of these regions received direct inputs from a much wider array of brain regions than previously appreciated. Many of these inputs were not detected by previous studies, as they may have escaped detection using less sensitive methods, or their relatively low abundance may have been ascribed to an error in injection targeting. It was also surprising that the VTA and SNc largely shared the same inputs, in largely the same proportions, with a few unique exceptions that include the motor cortices and subthalamic nucleus for the SNc and the lateral hypothalamus for the VTA. A similar approach has been used to examine the inputs to different groups of serotonergic neurons (Ogawa et al., 2014; Pollak Dorocic et al., 2014; Weissbourd et al., 2014), as well as the dorsal striatum (Wall et al., 2013) and hippocampus (Sun et al., 2014).

#### RABV Tracing During Development can Reveal the Timing of Synapse Formation

Viral tracing methods have also been used to investigate the timing of wiring and synapse formation during development. One such study investigated neurons potentially involved in rodent whisking behavior (Takatoh et al., 2013). The monosynaptic technique is particularly useful for this application, as it is able to identify *when* synaptic connections between neurons are formed, which would not be possible using classical tracers. In order to find such neurons, the investigators used the fact that whisking is initiated postnatally, and therefore examined inputs to the vibrissal motor neurons at different postnatal days, with the goal of finding populations of inputs that were specifically labeled *after* the onset of whisking. By comparing the label produced by injections at P1 and P8, the authors discovered new inputs from the rostral part of the lateral paragigantocellularis in the hindbrain that were identified during the later time window. As these neurons appear to get direct input from the motor cortex, the authors suggest that the formation of inputs from these hindbrain neurons is critical for active whisking. Another study used RABV to examine the formation of inputs onto newly generated neurons in the dentate gyrus in the hippocampus, and adult-born neurons in the olfactory bulb (Deshpande et al., 2013). In both cases, they found that at early time points post-maturation, the majority of the transsynaptically-labeled inputs were located nearby the cells from which tracing was initiated. If the researchers initiated tracing a few weeks after maturation, they observed the presence of an increasing number of inputs located at greater distances from the targeted neurons, suggesting that newly born neurons first integrate into local circuitry before they receive inputs from distant sites. The similar pattern of observations in both the hippocampus and the olfactory bulb may indicate a common mechanism for integrating newborn neurons into already-established neural networks.

## Viruses Engineered to Carry Functional Genes

In addition to the rapid development of transsynaptic viral technologies, in the past decade there has been a veritable explosion of genetically-encoded functional tools for measuring and manipulating neuronal activity (Fenno et al., 2011; Looger and Griesbeck, 2012; Farrell and Roth, 2013). These include, but are not limited to, genetically-encoded calcium and voltage indicators for imaging neuronal activity (Looger and Griesbeck, 2012) and optogenetic and pharmacogenetic effectors for manipulating neuronal activity (Fenno et al., 2011; Farrell and Roth, 2013). These new functional tools have already led to a plethora of circuit-level discoveries, primarily aided by transgenic strategies that target expression to specific cell-types in localized regions of the brain (Ting and Feng, 2013). In some cases, as in the Thy1 lines of transgenic mice, expression of these genes can be restricted to specific subsets of projection neurons, offering the possibility of pathway-specific functional analysis and behavioral readout (Arenkiel et al., 2007; Dana et al., 2014).

Ultimately, though, transgenic strategies alone are insufficient to target the many different cell-types in the brain that are currently defined not by their genetic profile, but by their unique pattern of connections with other neurons within and between different brain areas (Jones, 1984; Douglas and Martin, 2004). Recently, there has been a concerted effort to exploit the power of viral tracers in order to deliver these functional tools to neuronal populations defined by their connectivity. This has led to a revolution in efforts to directly relate structure to function, with enormous promise for model species such as the primate for which transgenic technology is not currently available (but see Kishi et al., 2014).

The viral tracing tools outlined in the previous section enable researchers to study anatomical circuits with unprecedented precision. However, the rapid gene expression from these vectors, which may be critical for their high efficiency of transsynaptic transfer, makes them disadvantageous for studying circuit function, as the speed of gene expression comes at the expense of neuronal health. This is usually not an issue for anatomical studies with relatively short survival times, but it poses a major challenge for functional studies, especially those that require chronic experimentation in behaviorally trained animals. Therefore, in order to deliver genes to defined neuronal classes in which long-term monitoring or manipulation is necessary, a different methodology and set of reagents is required.

To be useful for functional and behavioral experiments, it is essential that the virus of choice be able to deliver a high payload without harming the infected cells. This has motivated a variety of viral targeting strategies, the most successful of which have so far involved AAV, either alone or in combination with other viruses, due to its ability to express high levels of protein with relatively low levels of toxicity. In most cases, regardless of the specific functional tool or application, the relevant viral targeting strategies are similar. Some specific examples using these strategies have already been described in previous sections, so here we provide a broad overview of three main types of viral approaches that have been pursued to target the expression of genes to specific cell types for long-term analyses, highlighting previous and ongoing efforts to obtain a functional or behavioral readout of a specific pathway or circuit in the brain.

### Axon Terminals

**Figure 4:**
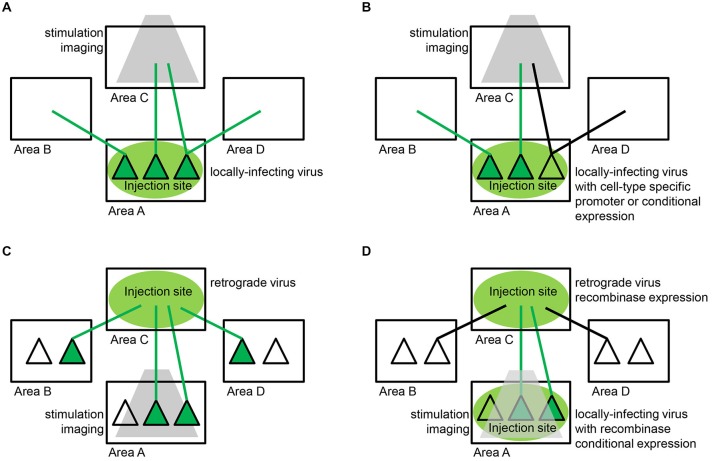
**Viral strategies for delivering functional tools to neuronal populations defined by their connectivity. (A)** Axon terminal targeting. The most successful approach to date relies on locally-infecting viruses, such as lentivirus and adeno-associated virus (AAV), which enable expression of a functional tool within neuronal cell bodies (dark green triangles) and dendrites local to the injection site (area A) and throughout their axons (green lines) that terminate both locally and in remote areas of the brain (areas B,C,D). Specific efferent pathways originating from the injection site are targeted by restricting stimulation or imaging (gray) to one particular target area (area C). **(B)** Cell-type specific axon terminal targeting. In some circuits, spatially-intermixed parallel pathways converge on the same target area of interest. In order to disentangle these pathways, the same locally-infecting viruses described in **(A)** can be engineered to target specific cell-types local to the injection site (area A). This can be accomplished with cell-type specific promoter sequences or recombinase-dependent expression sequences that restrict expression of a functional tool to a genetically-defined subpopulation (dark green triangles in area A). Specific efferent pathways originating from that particular subpopulation (green lines) and not other parallel pathways (black lines) are targeted as in **(A)** by restricting stimulation or imaging (gray) to one particular target area (area C). **(C)** Projection neuron targeting. Retrograde viruses, such as rabies, enable expression of a functional tool in specific projection neuron populations. Retrograde virus is taken up by axonal terminations (green lines) local to the injection site (area C) and travels back to cell bodies (dark green triangles) throughout the brain (areas A,B,D). Specific afferent pathways projecting to the injection site are targeted by restricting stimulation or imaging (gray) to one particular input area (area A). Retrograde viruses alone are often unable to achieve the high levels of expression needed for long-term functional studies. **(D)** Intersectional approaches. A combination of the strategies described in **(A,C)** above have enabled high levels of expression with low levels of toxicity in specific projection-neuron populations. The most successful strategy to date involves delivering small amounts of Cre-recombinase via a retrograde virus and also injecting a locally-infecting virus encoding Cre-dependent expression of a functional tool at the location where the targeted projection neurons originate. Specifically, retrograde virus is taken up by axonal terminations (green and black lines) local to the injection site (area C) and travels back to cell bodies throughout the brain (areas A,B,D). The retrograde virus expresses Cre-recombinase within these infected projection neurons. Specific afferent pathways (dark green triangles) projecting to the injection site are targeted by injecting a locally-infecting virus, such as AAV, encoding Cre-dependent expression of a functional tool in the target area of interest (area A). This strategy can functionally isolate neurons that reside in area A and project to area C, obviating the need for site-specific stimulation or imaging (gray).

The most successful integration of viruses and functional tools thus far has relied on a viral strategy that does not explicitly employ viruses as tracers, but, instead, simply depends on robust expression of a virus’s genetic payload throughout the soma, dendrites and, in particular, axons of neurons local to the injection site (Figure 4A). AAVs and lentiviruses have been the most commonly used viruses for purposes of local infection and robust expression throughout the axonal tree, with the important advantage of being chronically well-tolerated and minimally toxic (Osten et al., 2006; Büning et al., 2008). In this way, these viruses serve as a sort of anterograde tracer, enabling functional analysis of specific efferent pathways originating from neurons at the injection site (Figure 4A).

For example, in the case of optogenetics, light can be delivered specifically to a particular target area and activate only the axon terminals of the infected neuronal population, thus allowing the physiological and behavioral consequences of a particular set of inputs to be measured (Petreanu et al., 2007; Gradinaru et al., 2009; Cruikshank et al., 2010; Stuber et al., 2011). Such axonal stimulation can be used to map out how long-range connections in the brain interact with a local microcircuit (Petreanu et al., 2007) or, with the aid of functional magnetic resonance imaging (fMRI), across the entire brain (Lee et al., 2010; Desai et al., 2011).

For imaging applications, calcium levels within axons in a particular target area of the infected neuronal population can be measured (Reiff et al., 2010; Petreanu et al., 2012; Glickfeld et al., 2013). Note that in all of these cases, pathway specificity is not obtained by the virus alone, which will often infect a heterogeneous population of neurons projecting to many different brain regions. Rather, pathway specificity is achieved by combining the viral infection of the soma with spatial restriction of either the light beam used for stimulation or the field of view of the microscope to a brain region where only one particular projection pathway terminates (Figure 4A). In some circuits, however, spatially intermixed parallel pathways exist, requiring additional tricks to disentangle the functional influence of one pathway from another. Cell-type specific expression can sometimes aid in such efforts, either by using AAVs and lentiviruses encoding cell-type specific promoter sequences or by using transgenic lines with cell-type specific expression of Cre-recombinase and viruses with Cre-dependent expression sequences (Betley and Sternson, 2011; Figure 4B).

### Cell Bodies of Projection Neurons

While stimulation and imaging of axons has already led to great progress in the field, there are many instances in which it would be preferable to modulate or image the activity of specific projection neuron populations at their site of origin (Figure 4C). This is especially true when the axons of a particular pathway are difficult to access or when they are defined not simply by the brain area in which they terminate, but by the specific cell-type within that area onto which they synapse. In such cases, researchers have turned toward several of the retrograde viruses described previously, which travel back from axonal termination to the cell body. Similarly to the axonal stimulation strategy described above in the “Axon Terminals” Section, pathway specificity is obtained not by the retrograde virus alone, which will infect neurons projecting from many different brain regions, but by restricting the stimulation area of the light or the field of view of the microscope to a brain region where only one particular projection pathway originates (Figure 4C).

One of the most powerful viral tracers for these purposes is RABV, due to its exclusively retrograde spread, fast expression dynamics and high amplification of its genes (Ugolini, 1995; Kelly and Strick, 2000). Though not useful for chronic, longitudinal experiments because of its relatively fast-onset toxicity (cell death as early as 14 days post-infection), the SAD-B19-ΔG strain of rabies can be used in an acute setting to express high levels of light-sensitive channel or calcium indicator in specific projection neuron populations based on the target region injected with virus (Wickersham et al., 2007a; Osakada et al., 2011). It can also be complemented *in trans* to enable monosynaptic spread restricted to the inputs of an initially infected population of neurons (Wickersham et al., 2007b).

For chronic experiments, however, retrograde viruses with lower toxicity profiles are required. While potentially somewhat cytotoxic, genetically-modified retrograde viruses could potentially be used for these purposes, including Herpes Simplex Virus Type 1 (HSV-1), the Bartha strain of pseudorabies (PRV) or CAV (Kremer et al., 2000; Frampton et al., 2005; Tomioka and Rockland, 2006; Card and Enquist, 2014). Another possibility is to pseudotype viruses of low toxicity that do not normally move retrogradely, such as lentivirus, with RABV or RABV-VSV-G chimeric glycoproteins, so that they do in fact move retrogradely from the site of injection (Mazarakis et al., 2001; Kato et al., 2011). Ultimately, though, the expression levels produced by most of these retrograde viruses on their own are typically insufficient for functional efficacy. While certain genetic modifications might enable higher expression levels (Cetin and Callaway, 2014), it remains to be seen whether any low-toxicity, retrograde virus on its own can produce the high levels of expression needed for long-term chronic functional and behavioral studies.

### Intersectional Approaches

A combination of different viral approaches has recently shown promise as a way to achieve high levels of expression with low levels of toxicity in specific projection-neuron populations. The most successful strategy to date involves delivering small amounts of Cre-recombinase via a retrograde virus after injecting a locally-infecting AAV encoding Cre-dependent expression of a functional tool at the location where the targeted projection neurons originate (Figure 4D). This has already been successful with several retrograde viruses including HSV-1 (Lima et al., 2009; Senn et al., 2014), PRV (Granstedt et al., 2010; Chaudhury et al., 2013) and CAV-2 (Hnasko et al., 2006; Senn et al., 2014). The approach requires careful matching of the two injection sites in order to target the same projection population with both viruses. Non-viral retrograde delivery of Cre-recombinase, such as with the plant lectin WGA, is also an option (Gradinaru et al., 2010; Xu and Südhof, 2013), though bi-directional and diluted multi-synaptic spread of WGA may limit its utility (Köbbert et al., 2000).

These intersectional approaches have the important added advantage of specific expression in the particular projection neuron population of interest, rather than all populations that project to the injection site (Figure 4D). This, then, opens up the possibility of using systemic rather than local effectors such as in the case of tetracycline-controlled transcriptional activation (tet-ON and tet-OFF) or designer receptors exclusively activated by designer drugs (DREADDs; Mansuy and Bujard, 2000; Farrell and Roth, 2013). Anterograde viral strategies currently under development would likely benefit from a similar intersectional approach and would vastly expand the pathways and circuitry accessible to functional and behavioral analysis in future studies (Beier et al., 2011b; Lo and Anderson, 2011). Intersectional approaches for either anterograde or retrograde tracing also can be employed that use the overlap of expression patterns of Cre and Flp. These two recombinases can be expressed from transgenic configurations (Dymecki et al., 2010) and/or via viral delivery (Fenno et al., 2014). Use of an effector gene, such as TVA, that is dependent upon expression of both Cre and Flp creates greater specificity in the cell types being targeted for infection or viral spread. This type of intersectional approach will also benefit from a new method of manipulating GFP-expressing cells, referred to as “Cre-dependent upon GFP” (Cre-DOG) where GFP+ cells in a transgenic GFP line are induced to express Cre (Tang et al., in press). These GFP+/Cre+ cells can be intersectionally selected by expressing Flp from either a transgenic locus or a virally delivered Flp.

## Future Directions

The use of neurotropic viruses has vastly expanded the scope of neuroanatomy, and the genetic engineering of these viruses has permitted exciting new experiments in which functional studies are coupled to anatomy in a direct way that has revealed new insights into neural circuits. As the preceding sections have made clear, however, there remains room for improvement along several dimensions. In this section, we briefly consider several major obstacles and possible strategies for overcoming them.

### Reducing Toxicity

One of the major limiting factors for merging transsynaptic viral vectors with functional approaches is their rapid toxicity. Efforts are ongoing to reduce the toxicity of these transsynaptic vectors, largely by mutating viral proteins. For example, a point mutant in the VSV M protein (M51R) was used to permit slice recordings in VSV-infected neurons where VSV encoding the wild-type M protein was too toxic to permit physiological investigation of post-synaptic cells (Beier et al., 2011b). The M protein has been implicated in the persistence of infection (Ahmed and Lyles, 1997; Desforges et al., 2001) as well as other deleterious processes. There are several mutations in M that can be tested for their effects on toxicity and efficacy on transsynaptic tracing. As RABV has a similar set of genes as VSV, similar mutations might be profitably applied to these vectors as well. In addition, while RABV, VSV, and PRV/HSV have been used for controlled studies of transsynaptic transmission, synapse-restricted transmission may not be an exclusive property of these viruses. For example, the Borna virus (BDV) has been reported to have patterns of neuron-to-neuron spread consistent with transsynaptic transmission (Carbone et al., 1987; Morales et al., 1988; Gosztonyi et al., 1993). It may therefore be possible to engineer viruses that are inherently less toxic than those currently used to trace synaptic connections.

### Improving Control of Transsynaptic Spread

One of the major limitations of manipulating viruses for monosynaptic tracing is that the only two viruses thus far used for this purpose (RABV and VSV) are RNA viruses, and are therefore inaccessible to recombinase technology. Therefore, no methods currently exist to modify the virus once it is injected into the animal. However, it may be possible to directly modify viral RNA. For example, one could take advantage of the differential effects of three-dimensional structure on RNA function. Riboswitches are short sequences in messenger RNAs that bind small molecules, changing the RNA conformation and the levels of proteins translated from those RNAs (Vitreschak et al., 2004; Roth and Breaker, 2009). These can be screened or selected from a pool of synthetic libraries for binding to specific small molecules. Such RNA aptamers could be expressed from the viral genome, and could bind to a small molecule administered to the animal after the virus was injected. It may also be possible to express shRNAs or antisense transcripts within certain cell types that could either eliminate or slow viral replication and/or transcription within those cells. Also, we may be able to take advantage of the clues that Mother Nature has provided. The innate immune response, which limits the spread of VSV, but not RABV, from the periphery may provide a mechanism to limit spread once a virus enters the CNS. We will need to better understand the immune response to different transsynaptic viruses to exploit this system.

### Improving the Efficiency of Transsynaptic Spread

We currently have only a limited understanding of how transsynaptic spread occurs. It is not always appreciated that these viruses spread among non-neuronal cells *in vitro*. That is, synapses are not needed for infection or for transmission, and many different cell types can host virus replication. Studies of the mechanism(s) that limit spread to synaptically connected neurons *in vivo*, as well as govern the direction of spread, are needed to allow an improvement in efficiency, which is currently only 2–5% for retrograde labeling of presynaptic partners of VSV and RABV (Marshel et al., 2010; Beier et al., 2013a). In addition, the mechanisms that limit transmission in some species, such as Drosophila, require investigation.

### Extending the Range of Species that can be Studied

The vast majority of anatomical studies cited in this review were performed in mice. Mouse genetics have allowed for a great deal of flexibility in the design and execution of experiments that rely on the introduction of genes that direct infection and spread of the virus. However, it is clearly necessary to extend viral tracing methods to other organisms—ranging from flies to monkeys—both because such animals represent important model systems in their own right and because comparative studies are essential to investigate the evolution of circuits. One virus with the potential to infect a wide range of species is VSV, which was recently shown to infect neurons in animals ranging from invertebrates to nonhuman primates and to replicate and be transported in several important model organisms, including zebrafish and chicks (Mundell et al., 2015).

The current use of transsynaptic viral vectors is limited to the capabilities of the vectors used. That is, we currently have only a limited understanding of how viruses transmit among neurons in the CNS. While RABV, VSV, and HSV/PRV have all displayed the potential to transsynaptically label neurons in multiple species, this could be expanded by, for example, expressing the appropriate viral receptors in the host cells, and/or enabling replication in otherwise non-permissive cells. For example, VSV does not appear to replicate in Drosophila neurons as a result of an innate immune response that triggers autophagy (Shelly et al., 2009). Developing a more nuanced understanding of the mechanisms of viral infection, replication, and transmission at the basic science level will permit a whole new generation of transsynaptic techniques that have the potential to refine and enhance the capabilities of these methods.

## Closing Remarks

The considerations presented above remind us of the power, beauty and critical importance of research on the basic mechanisms of biology—even, or especially, of such seemingly exotic creatures as viruses. While some might believe that the main reason to study viruses is to create vaccines that limit their spread in human populations, it should be clear that we need to understand these remarkable entities in much more mechanistic detail so that we can engineer the next generation of neuroanatomical tools with which to solve the function of circuits within our brains and thus to tackle other devastating diseases—diseases of brain mis-wiring, such as autism (Minshew and Williams, 2007; Gepner and Féron, 2009) and schizophrenia (Rubinov and Bassett, 2011).

## Conflict of Interest Statement

The authors declare that the research was conducted in the absence of any commercial or financial relationships that could be construed as a potential conflict of interest.
